# Supplementary material for: Improving Vitamin D Intake in Young Children—Can an Infographic Help Parents and Carers Understand the Recommendations?
Source: Nutrients. 2021 Sep 9;13(9):3140. doi: 10.3390/nu13093140 (PMC8469200; doi:10.3390/nu13093140)
Supplement: Supplementary file 1 [file nutrients-13-03140-s001.zip › Moderators guide_supplementaryfile1.pdf]

LEEDS BECKETT UNIVERSITY
SCHOOL OF CLINICAL & APPLIED SCIENCES

Focus group/interview topic guide

**Introductory Comments:**

Hello, I'm Ailsa a student from Leeds Beckett University.

Thank you for coming today. As the information sheet explained, the purpose of this focus group is to help us gather your feedback about this infographic that we have designed to support families with their vitamin D intake.

The focus group/interview should take around 45 minutes, most likely less.

Before we start, I just need to remind you that taking part is entirely voluntary, you can leave at any time and if there are any questions that you don't want to answer that is ok.

Can I just check that you are still happy to take part?

We would like to record the focus group/ interviews to help us with accuracy. Is everybody ok with that? (if yes- start recording, if no- just take notes)

Can I also just check that you have had a chance to read the information sheet? Do you have any questions? If no - there is one here please read this and then sign the consent form

Can I now ask you to please read and complete this consent form and then also please fill out this short questionnaire

**If they have read the sheet:**

I just need to remind you that your answers will be anonymous

**If they have not read the information sheet:**

I need to let you know that anything you do say will be treated confidentially, your name will not be put with your responses and they are strictly confidential. This means that your name will not be put with anything you say. Any information you give us will be stored securely and only the research team at the university will have access to it. We will be producing a journal article with the findings, that will be shared with education and health professionals.

Today I've got some questions I'd like to ask you. If you could answer as fully as you can, that would be great.

At the end, there will be a chance for you to add any additional comments about the project.

Any questions for me before we start?

Ok, let's get started.

**Acceptability and perceptions of the infographic**

1. Overall, what do you think about the infographic? **(listen to interpretation)**
2. Is it clear what this information sheet (known as an infographic) is for? If yes why? If no why?*
3. What do you like most about it? Please explain
4. Are there any parts that you do not like or would like to change? Please give examples and why
5. Can you easily understand the specific information at the different stages?
6. Is there anything you don't understand about the information sheet? If yes please expand.

**Design / presentation**

1. What do you think about the presentation of the infographic (how it looks)? E.g. layout, language used, colours, pictures etc.*
2. Is anything missing that you would like to see in the layout of the infographic?
3. Is there anything more we could we do to improve the presentation of the infographic?*

**Content**

1. What do you think about the information about vitamin D provided on the infographic? *
2. What do you think about the language used i.e. the way it is written is easy to understand? *
3. Has it covered everything you would like it to cover? Anything missing?
4. Is there anything more we could do to improve the information on the infographic?*
5. Is it clear that this advice/information displayed is from a trustworthy and reliable source? If yes why? If no why?*

**Understanding**

1. Do you think other parents or pregnant women will understand the infographic? Is it easy to read/clear?
2. Are there any parts that need to be clearer? *
3. What do you think we could do to make it easier to understand for parents or pregnant women? *
4. How would you feel about following this advice and taking supplementation yourself and/or supplementing your child if appropriate?

**Suitability**

1. Do you think the infographic is suitable for pregnant mothers to help them with their vitamin D requirements? If yes, please explain why.  If no, how can we make it more suitable?*

2. Do you think the infographic is suitable for parents/caregivers to help them with their child's vitamin D requirements? If yes, please explain why? If no, how can we make it more suitable?*
3. What do you think about the different methods for increasing vitamin D intake presented? Are they clear? suitable/accessible (e.g. supplements or fortified foods)? easy to understand? *
4. Can you think of any challenges/ barriers preventing parents or pregnant women from using the infographic? (e.g. understanding, costs, accessibility etc.) What could we do to help overcome these challenges/barriers?

**Advertising**

1. Where would you expect to see this infographic?*
2. Where would you like to see this infographic? I.e. anywhere that would be helpful to you
3. If you were going to be given the infographic to take home, in what form would you like to receive it (i.e. email, printed copy, on social media)

**Interpretation and application of the infographic**

1. Are there any methods which you would not be happy to use for increasing vitamin D intake for yourself/during pregnancy/or for your child? (i.e. formula, supplementation) Please explain why?
2. Other than what is on the infographic, can you think of any other methods for increasing vitamin D intake for yourself/ your child/during pregnancy that you would use? (i.e. through food)

**Other**

1. Can you suggest anything else that you would like to see in the infographic? Website, contact, more info
2. Do you have any further comments you would like to add?

**Thank you so much for your time!**

**KEY:**

* = Potential repetitive question

Highlighted = selected questions transcribed
