# Supplementary material for: Improving Vitamin D Intake in Young Children—Can an Infographic Help Parents and Carers Understand the Recommendations?
Source: Nutrients. 2021 Sep 9;13(9):3140. doi: 10.3390/nu13093140 (PMC8469200; doi:10.3390/nu13093140)
Supplement: Supplementary file 1 [file nutrients-13-03140-s001.zip › Vitamin D Infographic supplementary file 2.pdf]

VITAMIN D

The Developmental Stages

Pregnancy

A vitamin D supplement containing 10 μg/day for mother DAILY

Breastfeeding

exclusively 0–6 months

A vitamin D supplement containing 10 μg/day for mother DAILY

Vitamin D supplement containing 8.5-10 μg/day DAILY for infants aged 0-1 years (providing they are not receiving more than 500ml infant formula daily)

Formula

feeding 0–6 months

Formula-fed babies should not be given a vitamin D supplement until they are having less than 500ml (about a pint) of infant formula a day, as infant formula is fortified with vitamin D

Weaning

6 months–1 year old

If child is receiving less than 500ml formula daily: vitamin D is recommended in the range 8.5-10 μg/day for ages 0 up to 1year

If child is breastfed (and receiving less than 500ml formula daily): vitamin D is recommended in the range 8.5-10 μg/day for ages 0 up to 1 year

Child

aged 1–4 years old

Vitamin D supplement containing 10 μg/day DAILY
